# Inferring the Timing of Antiretroviral Therapy by Zero-Inflated Random Change Point Models Using Longitudinal Data Subject to Left-Censoring

**DOI:** 10.3390/a18060346

**Published:** 2025-06-05

**Authors:** Hongbin Zhang, McKaylee Robertson, Sarah L. Braunstein, David B. Hanna, Uriel R. Felsen, Levi Waldron, Denis Nash

**Affiliations:** 1 Department of Biostatistics, College of Public Health, University of Kentucky, Lexington, KY 40506, USA; 2 Institute for Implementation Science in Population Health, City University of New York, New York, NY 10027, USA; 3 Bureau of Hepatitis, HIV, and Sexually Transmitted Infections, Department of Health and Mental Hygiene, New York, NY, USA; 4 Department of Epidemiology and Population Health, Albert Einstein College of Medicine, Bronx, NY 10461, USA; 5 Department of Medicine, Albert Einstein College of Medicine and Montefiore Medical Center, Bronx, NY 10461, USA; 6 Department of Epidemiology and Biostatistics, Graduate School of Public Health and Health Policy, City University of New York, New York, NY 10027, USA

**Keywords:** random change point model, zero-inflated exponential distribution, nonlinear mixed-effects model, censored data, Stochastic EM, Gibbs sampler, Metropolis–Hastings sampling, antiretroviral therapy

## Abstract

We propose a new random change point model that utilizes routinely recorded individual-level HIV viral load data to estimate the timing of antiretroviral therapy (ART) initiation in people living with HIV. The change point distribution is assumed to follow a zero-inflated exponential distribution for the longitudinal data, which is also subject to left-censoring, and the underlying data-generating mechanism is a nonlinear mixed-effects model. We extend the Stochastic EM (StEM) algorithm by combining a Gibbs sampler with a Metropolis–Hastings sampling. We apply the method to real HIV data to infer the timing of ART initiation since diagnosis. Additionally, we conduct simulation studies to assess the performance of our proposed method.

## 1. Introduction

Antiretroviral therapy (ART) is the cornerstone of HIV care, and timely initiation is critical for improving clinical outcomes and reducing HIV transmission. While current guidelines endorse immediate ART initiation upon diagnosis, in practice, the time between HIV diagnosis and treatment initiation varies considerably. These delays often arise due to multiple barriers, including anticipated side effects (i.e., perceived collateral effects of ART), stigma, mental health or substance use challenges, and structural issues such as inconsistent access to care. As a result, viral load trajectories observed in clinical data reflect substantial heterogeneity in ART uptake timing.

While HIV case surveillance in most U.S. states collects viral load data through mandatory electronic reporting, information on ART initiation is typically not recorded. Public health departments often infer treatment initiation indirectly using viral suppression data or through analyses of clinical cohorts. Motivated by Braunstein et al. [1], who developed a rule-based method using viral load surveillance data to estimate ART initiation timing in New York City, this paper proposes a statistical modeling approach to infer ART initiation time more formally and flexibly.

Random change point models, allowing for individual-specific changes in longitudinal outcomes, are widely used in medical research [2]. Typically, these models assume a normal distribution for the change point and use linear mixed-effects models for the segments before and after the change point [3–6]. However, the Gaussian assumption may not always be ideal. In our case, many individuals likely initiated ART at the time of diagnosis (time zero), making a zero-inflated distribution more suitable. Additionally, longitudinal data may be censored, meaning measurements below a certain threshold cannot be accurately quantified. For viral load data, the detection limit is generally between 50 copies/mL and 400 copies/mL. A linear model based on the observed data might not be suitable for the unobserved data. Alternatively, if a mechanical or scientific model is available for the longitudinal data, it can provide better predictions for the unobserved data and improve change point estimation. Such a mechanical model often takes a nonlinear form.

Accounting for a random change point within a likelihood framework poses a major challenge due to the lack of closed-form expressions [7–9]. This challenge is compounded by the non-Gaussian distribution of the change point, nonlinear models, and data censoring. In this paper, we propose a zero-inflated exponential (ZIE) distribution-based random change point model with segmented NLME sub-models to analyze left-censored data. To facilitate full likelihood-based inference, we extend the Stochastic Expectation-Maximization (StEM) algorithm, which was initially introduced by [10]. Our extension of the StEM involves a Gibbs sampler coupled with Metropolis–Hasting sampling for mixed-type random effects structure in the random change point model.

The remainder of this paper is structured as follows. Section 2 introduces the ZIE-based nonlinear random change point model. Section 3 presents the general model and details of the extended StEM algorithm. Section 4 analyzes an HIV cohort dataset, and Section 5 evaluates the proposed method through simulations. Finally, Section 6 concludes the article with a discussion.

## 2. ZIE-Based Nonlinear Random Change Point Model

In longitudinal data analysis, understanding how a specific outcome changes over time is crucial. Random effects change point models offer a valuable framework for examining changes in time trajectories by incorporating individual change points. These changes are typically induced by external events, causing deviations from the original data pattern.

Our objective is to determine when individuals initiate HIV treatment using their HIV viral load measurements over time. Without ART, the viral load fluctuates significantly after HIV infection until it stabilizes at a set point. If untreated, the viral load eventually increases, leading to AIDS [11]. Starting ART, however, results in substantial decreases in HIV viral load. To simplify our modeling approach, we assume the HIV diagnosis occurs after the viral set point and focus on modeling the changes in viral load dynamics post-ART initiation.

Traditional random effects change point models often assume that the longitudinal outcome can be described by a segmented linear mixed-effects model. However, as mentioned in the introduction, linear assumptions may not be suitable for many real-world applications. While linear models might adequately fit the observed data, they may not be appropriate for data subject to censoring, which is common with HIV viral load measurements, especially after ART initiation.

Extensive research has been conducted to understand the dynamics of viral load following ART initiation and to evaluate the effectiveness of these drugs in treating HIV. Building upon biological and clinical knowledge, ref. [12] proposed a virological model to approximate the patterns observed in viral load data. This model is represented by the equation:

$$V\left(t\right)=P_{1}e^{-\gamma t}+P_{2},$$

where V(t) represents the total virus at time t, and $P_{1}$ and $P_{2}$ are baseline values. The parameter γ corresponds to the viral decay rate and can be interpreted as the turnover rate of productively infected cells and long-lived or latently infected cells in an ideal therapy setting. Refs. [13,14] provide detailed discussions of this model.

For our problem, we can consider the following random change point model to describe the viral loads $y_{ij}$ for individual i at $j^{th}$ visit with time $t_{ij}$ after HIV diagnosis:

(1) $$y_{ij}=a_{1i}{\left(t_{ij}-\tau_{i}\right)}^{-}+{\text{log}}_{10}\left(b_{1i}e^{-b_{2i}{\left(t_{ij}-\tau_{i}\right)}^{+}}+b_{3i}\right)+e_{ij}.$$

Here, $y_{ij}$ is a log 10-transformed viral load, $\tau_{i}$ is the (single) change point which induces the change of viral load trajectory, and $e_{ij}$ is the error term. We use ${\text{log}}_{10}$ transformation in line with standard practice in HIV clinical research and surveillance, where viral load thresholds and treatment response metrics (e.g., a 1-log reduction) are conventionally interpreted on the ${\text{log}}_{10}$ scale. While natural logarithms are common in general modeling, the base-10 scale facilitates direct clinical relevance and comparability with prior studies.

The functions $x^{-}$ and $x^{+}$ correspond to min(x,0) and max(x,0), respectively. The quantity $a_{1i}$ represents the subject-specific regression coefficient that captures the viral load slope before the change point, while $b_{1i},b_{2i}$, and $b_{3i}$ represent subject-specific mixed effects governing the viral trajectory after the change point. We define these as

$$a_{1i}=\alpha_{1}+\alpha_{1i},\;b_{1i}=\beta_{1}+\beta_{1i},\;b_{2i}=\beta_{2}+\beta_{2i},\;b_{3i}=\beta_{3}+\beta_{3i}.$$

Here, $\alpha_{1},\beta_{1},\beta_{2}$, and $\beta_{3}$ represent the population parameters (fixed effects), while $\alpha_{1i},\beta_{1i},\beta_{2i}$, and $\beta_{3i}$ denote the random effects, typically assumed to follow normal distribution with a mean of zero. It is worth noting that the pre-change point segment and the post-change point segment meet at the intercept ${\text{log}}_{10}\left(b_{1i}+b_{3i}\right)$ when $t_{ij}=\tau_{i}$.

The choice of distribution for the random change points is a modeling assumption that depends on the specific investigation. For our application, there could be a significant proportion of individuals who presumably received ART treatment at diagnosis, i.e., “test-and-treated”. The rest would initiate their HIV treatment after the diagnosis date. The zero inflation exponential (ZIE) distribution allows the combination of a point mass at zero with an exponential distribution for the positive values [15]. It assumes that with a probability of π, the only possible observation is 0, and with a probability of 1−π, an exponential random variable is observed. For our change point model, we have

$$\tau_{i}\sim \left\{\begin{array}{ll} 0, & \pi \\ \text{Exp}(\lambda ), & 1-\pi \end{array}\right.$$

where λ represents the expectation of exponential distribution.

## 3. Estimation Procedure Based on StEM

### 3.1. The Models and Notations

In this section we describe the models and methods in a general form to illustrate their applicability to other applications. Let $y_{ij}$, with $j=1,2,\ldots ,n_{i}$, represent the longitudinal measurements for subject i=1,2,…,n, taken at time $t_{ij}$. We consider a general ZIE-based nonlinear random change point model:

(2) $$y_{ij}=g\left({\left(t_{ij}-\tau_{i}\right)}^{-},a_{i}\right)+h\left({\left(t_{ij}-\tau_{i}\right)}^{+},b_{i}\right)+e_{ij},\;a_{i}\sim N(\alpha ,A),\;b_{i}\sim N(\beta ,B),\;\tau_{i}\sim \text{ZIE}(\pi ,\lambda ),\;e_{ij}|a_{i},b_{i},\tau_{i}\sim N\left(0,\sigma_{e}^{2}\right).$$

Here, g(⋅) and h(⋅) are known nonlinear functions, and α and β are vectors of population parameters. The random effects $a_{i}$ and $b_{i}$ follow normal distribution with mean α and β, and covariance matrices A and B, respectively. The random change point $\tau_{i}$ is assumed to follow a zero-inflated exponential distribution with parameters π and λ. The within-individual variance is denoted as $\sigma_{e}^{2}$. The function $x(\cdot {)}^{-}$ and $x(\cdot {)}^{+}$ are defined as before. We assume that $a_{i},b_{i}$ are independent and both are independent of $\tau_{i}$, which is introduced externally.

To estimate and infer the model (2) for left-censored data, we employ a likelihood-based estimation procedure using the observed data $\left\{\left(q_{ij},c_{ij}\right),i=1,\ldots ,n,j=1,\ldots ,n_{i}\right\}$ where $c_{ij}$ is the censoring indicator such that $y_{ij}$ is observed if $c_{ij}=0$ and $y_{ij}$ is left-censored if $c_{ij}=1$. That is, $y_{ij}=q_{ij}$ if $c_{ij}=0$ and $y_{ij}\leq q_{ij}(\equiv d)$ if $c_{ij}=1$, where d is the detection limit. Extension to the “doubly-censored” case, in which the response may either be left-censored or right-censored, is straightforward.

Let $\theta =\left(\alpha ,\beta ,A,B,\sigma_{e}^{2},\pi ,\lambda \right)$ denote the collection of all unknown parameters, and let f(⋅) be a generic density function, with f(X∣Y) denoting the conditional density of X given Y. The observed data likelihood is given by the following:

(3) $$L\left(\theta \right)=\overset{n}{\underset{i=1}{\prod }}\left\{\int \;\int \;\int \left[\overset{n_{i}}{\underset{j=1}{\prod }}f{\left(y_{ij}|a_{i},b_{i},\tau_{i};\theta \right)}^{1-c_{ij}}\right.\right.\left.F{\left(d|a_{i},b_{i},\tau_{i};\theta \right)}^{c_{ij}}\right]\left.\times f\left(a_{i}\right)f\left(b_{i}\right)f\left(\tau_{i}\right)da_{i}db_{i}d\tau_{i}\right\},$$

where $F\left(d|a_{i},b_{i},\tau_{i};\theta \right)=\int_{-\infty }^{d}f\left(y_{ij}|a_{i},b_{i},\tau_{i};\theta \right)dy_{ij}$. Directly maximizing the likelihood (3) is challenging due to the presence of mixed-type distributions, nonlinear models, and nested integrals. The numerical methods, e.g., Gauss–Hermite Quadrature [16], can be prohibitively intensive for the computation. We therefore resort to EM algorithm-based methods. By treating $y_{\text{cen},i}$, the censored component of $y_{i}$, and the unobserved random effects $a_{i},\;b_{i}$, and $\tau_{i}$ as “missing data”, we have the “complete data” $\left\{\left(y_{i},a_{i},b_{i},\tau_{i}\right),i=\right.1,\ldots ,n\}$. The complete-data log-likelihood function for individual i is expressed as

(4) $$l_{c}^{\left(i\right)}(\theta )=\text{log}\;f\left(y_{i}|a_{i},b_{i},\tau_{i};\theta \right)+\text{log}\;f\left(a_{i};A\right)+\text{log}\;f\left(b_{i};B\right)+\text{log}\;f\left(\tau_{i};\theta \right).$$

### 3.2. The Estimation Procedure

The EM algorithm introduced by [17] is a classical approach to estimate parameters of models with non-observed or incomplete data. Let us briefly cover the principle. Denote z as the vector of non-observed data, (y,z) the complete data, and $L_{c}(y,z;\theta )$ the log-likelihood of the complete data; the EM algorithm maximizes the $Q\left(\theta |\theta^{\prime }\right)=E\left(L_{c}(y,z;\theta )|y;\theta^{\prime }\right)$ function in two steps. At the $k^{\text{th}}$ iteration, the E-step is the evaluation of $Q^{\left(k\right)}(\theta )=Q\left(\theta |\theta^{\left(k-1\right)}\right)$, where the M-step updates $\theta^{\left(k-1\right)}$ by maximizing $Q^{\left(k\right)}(\theta )$.

For cases where the E-step has no analytic form, ref. [18] introduces the MCEM algorithm, which calculates the conditional expectations at the E-step via many simulations within each iteration and hence is quite computationally intensive. The choice of replicate size is the central issue in guaranteeing convergence. Ref. [10] introduces a stochastic version of the EM algorithm, namely the StEM, which replaces the E-step with a single imputation of the complete data and then averages the last batch of M estimates in the Markov Chain iterative sequence to obtain the point estimate of the parameters. The imputed data $z^{\left(k\right)}$ at the $k^{\text{th}}$ iteration are a random draw from the conditional distribution of the missing data given the observed data and the estimated parameter values at the $(k-1{)}^{th}$ iteration, $f\left(z^{\left(k\right)}|y,\theta^{\left(k-1\right)}\right)$. As $z^{\left(k\right)}$ only depends on $z^{\left(k-1\right)},\;{\left\{z^{\left(k\right)}\right\}}_{k\geq 1}$ is a Markov chain. Assuming that $z^{\left(k\right)}$ take values in a compact space and the kernel of the Markov chain is positive continuous for a Lebesgue measure, the Markov chain is ergodic, and that ensures the existence of a unique stationary distribution [19,20].

In extending the StEM algorithm for the ZIE-based nonlinear random change point model, the imputation step is a crucial part of the process. At the $k^{\text{th}}$ iteration, we aim to draw the missing data $\left(y_{\text{cen},i^{\prime }}^{\left(k\right)}a_{i}^{\left(k\right)},b_{i}^{\left(k\right)},\tau_{i}^{\left(k\right)}\right)$, where direct sampling from the joint conditional distribution is often intractable. To address this, we employ a Metropolis-within-Gibbs sampler, wherein each component is updated conditionally. For variables with tractable full conditional distributions (e.g., censored outcomes), we use standard Gibbs updates. For components lacking closed-form conditionals, we embed Metropolis–Hastings steps within the Gibbs framework to sample from the appropriate target distributions. Unlike the EM or MCEM algorithms, this procedure does not require monotonic increases in the likelihood, but instead ensures that the Markov chain explores the parameter space in a way consistent with the joint posterior distribution [21,22].

As an example, after initializing $\theta^{\left(0\right)}$, and $\left(y_{\text{cen},i}^{\left(0\right)}{,a}_{i}^{\left(0\right)},b_{i}^{\left(0\right)},\tau_{i}^{\left(0\right)}\right)$, we update $y_{\text{cen},i}^{\left(k\right)},k=1,\ldots$, as follows:

Step 1: simulate $y_{\text{cen},i}^{*}$ from $\text{TN}(d,\mu_{i}^{\left(k-1\right)},\Sigma_{i}^{\left(k-1\right)})$, a multivariate truncated normal distribution with
  - d the lower bound of the truncation,
  - mean $\mu_{i}^{\left(k-1\right)}=(\mu_{i,1}^{\left(k-1\right)},\ldots ,\mu_{i,n_{i}}^{\left(k-1\right)})$ where $\mu_{ij}^{\left(k-1\right)}=g({\left(t_{ij}-\tau_{i}^{\left(k-1\right)}\right)}^{-},a_{i}^{\left(k-1\right)})+h\left({\left(t_{ij}-\tau_{i}^{\left(k-1\right)}\right)}^{+},b_{i}^{\left(k-1\right)}\right),j=1,\ldots ,n_{i}$,
  - and variance $\Sigma_{i}^{\left(k-1\right)}=\sigma_{e}^{2^{\left(k-1\right)}}I_{n_{i}\times n_{i}}$, where $I_{n_{i}\times n_{i}}$ is a $n_{i}$ by $n_{i}$ identity matrix, and, independently, sample η from the uniform (0, 1) distribution;
Step 2: calculate $\rho =\frac{l_{c}^{\left(i\right)}\left(\theta^{\left(k-1\right)}|\left(y_{\text{cen},i}^{*},y_{obs,i},a_{i}^{\left(k-1\right)},b_{i}^{\left(k-1\right)},\tau_{i}^{\left(k-1\right)}\right)\right)}{l_{c}^{\left(i\right)}\left(\theta^{\left(k-1\right)}|\left(y_{\text{cen},i}^{\left(k-1\right)},y_{obs,i},a_{i}^{\left(k-1\right)},b_{i}^{\left(k-1\right)},\tau_{i}^{\left(k-1\right)}\right)\right)}$;
Step 3: if η≤ρ, we update $y_{\text{cen},i}^{\left(k\right)}$ by $y_{\text{cen},i}^{*}$; otherwise, $y_{\text{cen},i}^{\left(k\right)}=y_{\text{cen},i}^{\left(k-1\right)}$.

The *maximization step* of the StEM algorithm involves maximizing the log-likelihood $\sum_{i=1}^{n}l_{c}^{\left(i\right)}\left(\theta |y_{\text{obs},},y_{\text{cen},i},a_{i},b_{i},\tau_{i}\right)$ to update parameters $\left\{\alpha ,\beta ,A,B,\sigma_{e}^{2},\pi ,\lambda \right\}$ under the current imputed missing data. Since $\left(y_{\text{cen},i},a_{i},b_{i},\tau_{i}\right)$ are regarded as data, the complete log-likelihood no longer involves integrals, which substantially simplifies the maximization. Solving the corresponding score functions yields the following estimations:

$$\alpha =\frac{1}{n}\sum_{i=1}^{n}a_{i},\;\beta =\frac{1}{n}\sum_{i=1}^{n}\;b_{i},$$

$$A=\frac{1}{n}\sum_{i=1}^{n}{\left(a_{i}-\alpha \right)}^{T}\left(a_{i}-\alpha \right),\;B=\frac{1}{n}\sum_{i=1}^{n}{\left(b_{i}-\beta \right)}^{T}\left(b_{i}-\beta \right),$$

$$\sigma_{e}^{2}=\frac{1}{n}\sum_{i=1}^{n}\frac{1}{n_{i}}\sum_{j=1}^{n_{i}}{\left\{y_{ij}-g\left({\left(t_{ij}-\tau_{i}\right)}^{-},a_{i}\right)-h\left({\left(t_{ij}-\tau_{i}\right)}^{+},b_{i}\right)\right\}}^{2}.$$

The likelihood function represented by the joint probability density and mass function of the ZIE distribution can be written as $L\left(\tau_{1},\tau_{2},\ldots ,\tau_{n};\pi ,\lambda \right)=\prod_{\tau_{i}=0}\pi \prod_{\tau_{i}>0}(1-\pi )\lambda \;\text{exp}\left(-\lambda \tau_{i}\right)$. Denote $I=\left\{\begin{array}{ll} 1, & \text{if}\;\tau_{i}=0\\ 0, & \text{other} \end{array}\right.$, we have $\text{log}\;L=\sum_{i=1}^{n}I\;\text{log}(\pi )+\sum_{i=1}^{n}(1-I)\;\text{log}\left[(1-\pi )\lambda \;\text{exp}\left(-\lambda \tau_{i}\right)\right]$. Solving the score function ∂logL/∂π=0 and ∂logL/∂λ=0, yields

$$\pi =\frac{1}{n}\sum_{i=1}^{n}I,\;\lambda =\left(1-\sum_{i=1}^{n}I\right)/\left(\sum_{i=1}^{n}\tau_{i}\right).$$

In general, increasing the number of random effects does not substantially increase the complexity of the maximization step. However, the imputation step will be more complicated, as this increases the dimensions of missing data that need to be imputed.

As with the likelihood defined in (3), the Fisher information matrix of the ZIE-based nonlinear random change point model has no closed-form solution. To obtain the variance-covariance matrix of the MLE $\overset{ˆ}{\theta }$, we consider the following approximate formula in [23]. Denote the score function of the complete-data likelihood by $S_{c}^{\left(i\right)}=\partial l_{c}^{\left(i\right)}/\partial \theta$. Then, an approximate formula for the variance-covariance matrix of $\overset{ˆ}{\theta }$ is

$$\text{Cov}(\overset{ˆ}{\theta })={\left[\sum_{i=1}^{n}\text{E}\left(S_{c}^{\left(i\right)}|y_{i},a_{i},b_{i},\tau_{i};\overset{ˆ}{\theta }\right)\text{E}{\left(S_{c}^{\left(i\right)}|y_{i},a_{i},b_{i},\tau_{i};\overset{ˆ}{\theta }\right)}^{T}\right]}^{-1},$$

where the expectation can be approximated by conditional mean of the Monte Carlo samples.

### 3.3. Convergence Diagnosis

Determining convergence is a critical aspect of the StEM algorithm, yet it remains an open question in the literature. The most commonly used approach for convergence diagnostics involves visual examination of trace plots [24–26]. Recently, ref. [27] proposed a Geweke Statistics-based method. We adopt this approach in our implementation for a more rigorous assessment of convergence. Specifically, for each run, after initializing the Markov chain with the specified initial values, we determined stationarity using a batch procedure based on the Geweke statistic [28]. A Geweke statistic is computed at each increment of w iterations using a moving window with batch size M. Specifically, the procedure consists of the following steps:

1. Initialization. Set B=0 and run the StEM algorithm to obtain the initial series of the estimates $\left\{\theta^{\left(wB+1\right)},\ldots ,\theta^{\left(wB+M\right)}\right\}$.
2. Check stationarity. For each entry p in θ, compute the Geweke statistic $z_{p}$ from the Markov chain $\left\{\theta_{p}^{\left(wB+1\right)},\ldots ,\theta_{p}^{\left(wB+M\right)}\right\}$. The Geweke statistic is defined as the standardized mean difference between the first $p_{1}$ and last $p_{2}$ portion of the chain, where $p_{1}$ and $p_{2}$ can be fine-tuned for a specific application. We consider stationary to be reached when all $\left|z_{p}\right|s$ are sufficiently small, i.e., $\sum_{p=1}^{P}z_{p}^{2}<\epsilon P$, where P is the total number of parameters and ϵ is another tuning parameter.
3. Update. If stationarity is not reached, perform w additional runs of the chain, increase the number B by 1, and repeat step 2.

## 4. Data Analysis

The HIV clinical cohort database (HCCD), maintained by Einstein-Rockefeller-CUNY Center for AIDS Research, contains de-identified data on people living with HIV and receiving care at hospitals and clinics affiliated with Montefiore Medical Center, which is the largest provider of HIV care in the Bronx, New York City. Patients in the HCCD are demographically representative with respect to age, sex, race/ethnicity, and HIV transmission risk of the overall population of people living with HIV in the Bronx that is described by public health surveillance data [29].

For this study, we include all patients living with HIV in HCCD diagnosed between 2005 and 2015 with last follow-up by 31 December 2017. Additional inclusion criteria included age ≥ 13 years and at least two HIV-1 viral loads recorded during the period. The final analytic dataset contains 2475 persons with a median viral load frequency of 5 and an inter-quantile range from 3 to 11. Notably, approximately 60% of the viral load measurements were found to be below the detection limits.

In addition to the primary model (2), we also fit a model where the post-ART segment is assumed to follow a two-compartment model [12], which is commonly used for viral load after the treatment. Therefore, we have the following two random change point models:

$\text{M1}:\;y_{ij}=a_{1i}{\left(t_{ij}-\tau_{i}\right)}^{-}+{\text{log}}_{10}(b_{1i}e^{-b_{2i}{\left(t_{ij}-\tau_{i}\right)}^{+}}+b_{3i})+e_{ij}.$
$\text{M2}:\;y_{ij}=a_{1i}{\left(t_{ij}-\tau_{i}\right)}^{-}+{\text{log}}_{10}(b_{1i}e^{-b_{2i}{\left(t_{ij}-\tau_{i}\right)}^{+}}+b_{3i}e^{-b_{4i}{\left(t_{ij}-\tau_{i}\right)}^{+}})+e_{ij}.$

where M1 allows for individual-specific baseline value $b_{3i}$ for the second phase of viral decay, while M2 also captures the second phase viral decay rate through the random effects $b_{4i}$. Our preliminary analysis indicates that it is sufficient to model the pre-change point segment with a linear mixed-effects model and the post-change point segment with a diagonal variance-covariance random effects structure. As a result, we make use of the following assumptions:

$\text{M1}:\;a_{i}\sim N\left(\alpha ,\sigma_{A}^{2}\right),\;b_{i}\sim N\left({\left(\beta_{1},\beta_{2},\beta_{3}\right)}^{T},\text{diag}\left(\sigma_{B11}^{2},\sigma_{B22}^{2},\sigma_{B33}^{2}\right)\right),\tau_{i}\sim \text{ZIE}\left(\pi ,\lambda \right),\;e_{ij}\sim N\left(0,\sigma_{e}^{2}\right).$
$\text{M2}:\;a_{i}\sim N\left(\alpha ,\sigma_{A}^{2}\right),\;b_{i}\sim N\left({\left(\beta_{1},\beta_{2},\beta_{3},\beta_{4}\right)}^{T},\text{diag}\left(\sigma_{B11}^{2},\sigma_{B22}^{2},\sigma_{B33}^{2},\sigma_{B44}^{2}\right)\right),\tau_{i}\sim \text{ZIE}(\pi ,\lambda ),\;e_{ij}\sim N\left(0,\sigma_{e}^{2}\right).$

We implemented the StEM algorithm in R [30] with the following tuning parameters: $\left(w,M,p_{1},p_{2},\epsilon \right)=(10,300,0.1,0.5,1.5)$, where we also restrict $\beta_{1}>\beta_{3}$ for M2 to ensure the model is identifiable. This configuration typically allows for convergence within 3000 iterations in most model-fitting runs. Owing to the stochastic nature of the StEM algorithm, the choice of starting values is quite flexible, with initial values set randomly within the possible range.

To simulate the multivariate truncated normal distribution for the left-censored viral loads, we utilized the R package truncnorm [31]. For the ZIE-distributed change points, we set the value to zero with the current estimated probability π, and with probability 1-π, we set it to a random variable from an exponential distribution with the current estimated mean λ.

**Figure 1.**
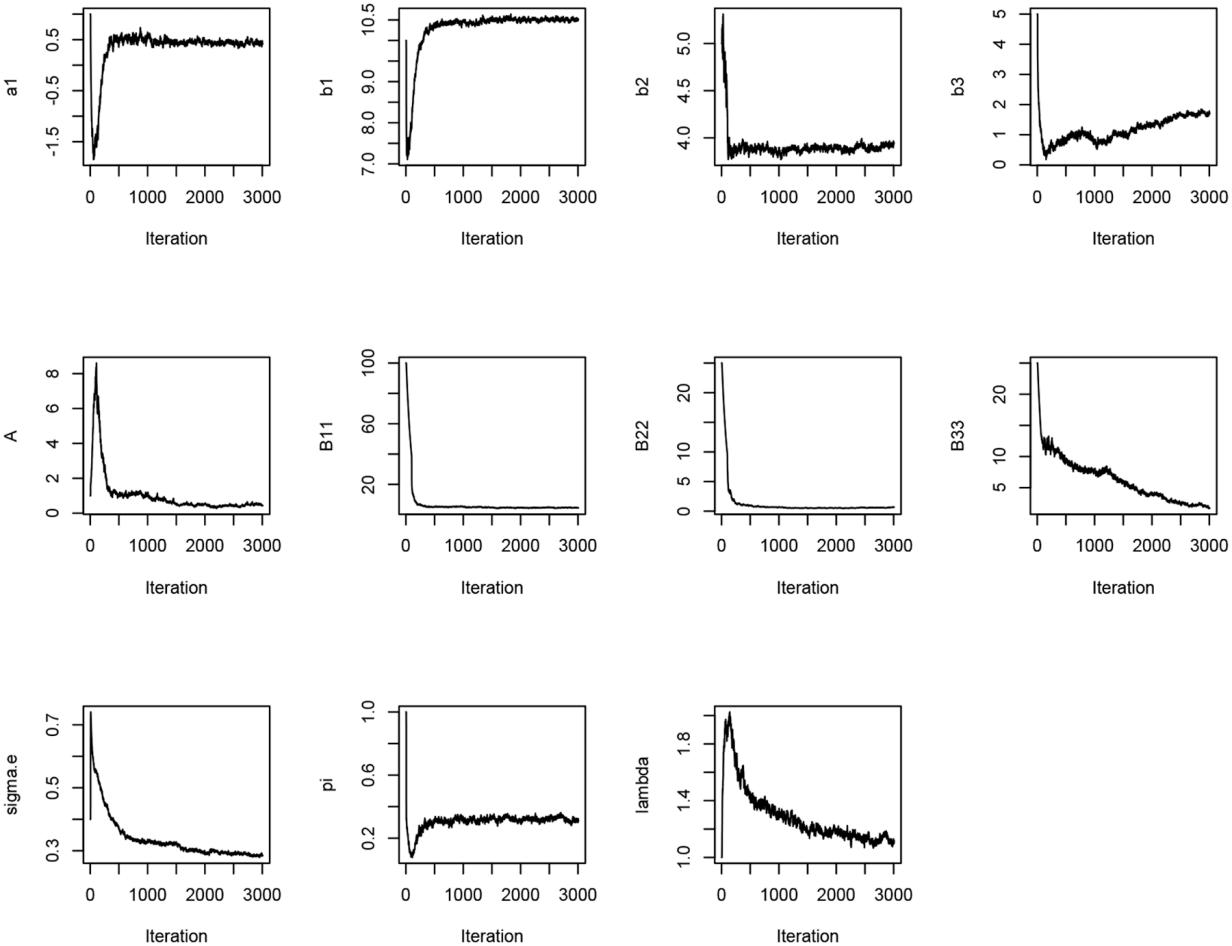
Trace plots for all parameters in M1.

**Figure 2.**
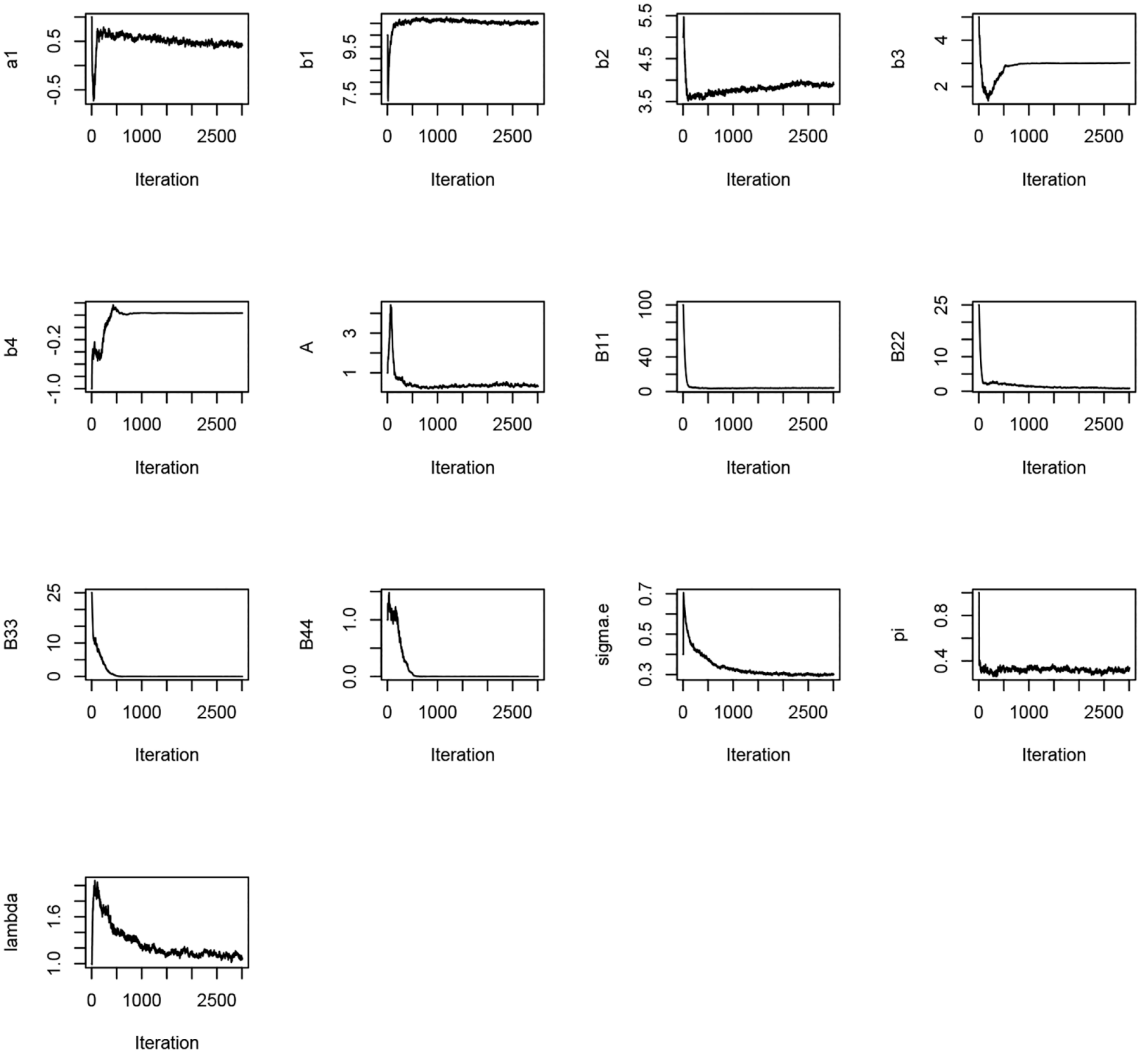
Trace plots for all parameters in M2.

Figures 1 and 2 display the trace plots of the Markov Chains for each parameter under models M1 and M2, respectively. Table 1 summarizes the estimation results for the fixed effects and dispersion parameters for both models. The estimations are comparable between M1 and M2, except for $\beta_{3}$ which is necessarily smaller in M1 due to the single baseline parameter in the one-compartment model M1. In contrast, this baseline value is estimated to be larger in M2, where it, along with the viral decay rate $\beta_{4}$, captures the dynamics of the viral trajectory during the second phase. Both M1 and M2 estimate a similar positive slope of viral load before ART initiation, specifically 0.43 and 0.42, respectively. Additionally, the models estimate similar percentages for the proportion of individuals who started ART treatment at time zero (time of diagnosis), with M1 estimating 31% and M2 estimating 32%.

We also assess the performance of our model by comparing the observed viral load values with the predicted values for each individual in the HCCD. Individual random effects, including the change point, are predicted using the conditional mean, which is obtained by averaging the parameters from extra iterations of the imputation step after convergence.

To further contextualize our model-based estimates, we also implemented the approach denoted as ${\text{log}1\text{plus}}^{*}$, adapted from the rule-based algorithm developed by [1], which uses viral load surveillance data to infer ART initiation timing. Specifically, ${\text{log}1\text{plus}}^{*}$ detects ART initiation when there is a decline of more than one ${\text{log}}_{10}$ unit in viral load between two consecutive measurements occurring within a defined time window (e.g., three months). Additionally, ART initiation is inferred when a subject transitions from being detectable to being undetectable (i.e., left-censored measurement). While the original method was primarily used to identify ART initiation in a subset of individuals affected by the treatment, ${\text{log}1\text{plus}}^{*}$ generalizes this logic to the entire sample. Furthermore, instead of imputing ART initiation at the midpoint between the two relevant viral load measurements, ${\text{log}1\text{plus}}^{*}$ assigns the initiation time to the earlier measurement in the pair. This adjustment is biologically motivated, reflecting the expectation that viral load suppression begins shortly after ART initiation.

**Figure 3.**
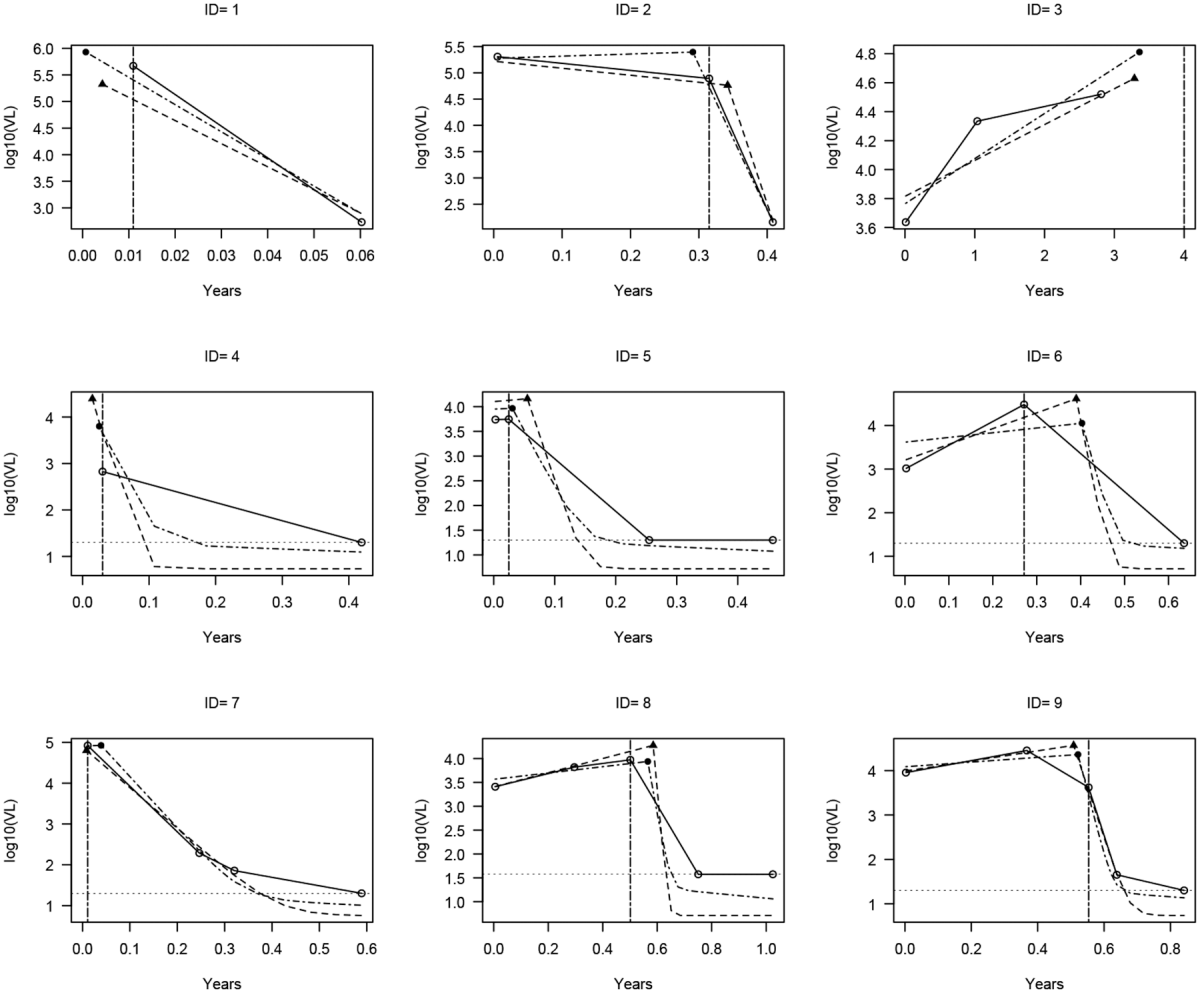
Individual trajectory prediction plots. Open circles (◦) connected by a solid line represent the observed viral loads. Dashed lines and the dash-dot lines indicate the predicted trajectory from M1 and M2, respectively. The detection limit (for IDs 4–9) is shown by dotted line. A triangle (▲) marks the predicted ART initiation time and corresponding viral load under M1, while a solid circle (•) does so for M2. The vertical dash-dot line represents the ART initiation selected by the ${\text{log}1\text{plus}}^{*}$ method. Visual comparison shows that this method often yields similar estimates to model-based predictions when there is a clear viral load decline, but may differ in cases with sparse or censored data (e.g., ID 6 or ID 8), highlighting the value of a model-based approach.

Figure 3 showcases the findings for nine selected individuals, chosen to represent typical patterns. For each individual, we present the predicted trajectory from M1 and M2, in addition to the observed viral loads. The predicted change point and the corresponding viral load at the time of ART treatment are indicated on the plot with different symbols for the two models. Predicted ART initiation by the ${\text{log}1\text{plus}}^{*}$ is also highlighted in the plot. It is important to note that the trajectories produced by the two models exhibit slightly different trends, with some trajectories displaying censoring (IDs 4 to 9) and others without censoring (IDs 1, 2, and 3).

Our model fits the fully observed data quite well and predicts a reasonable ART initiation time. For example, the change point is estimated right before the decline of the viral load for ID 1, while for ID 3, the change point is estimated beyond the last observed viral load, where the observed viral loads are ever-increasing, indicating no ART initiation. For ID 2, the ART initiation time occurs at about the time when the viral load starts to decline.

In contrast to ID 1, ID 4 has only one fully observed viral load, as the other is left-censored at around 4.8 months (0.4 years). Based on this information, M1 predicts that the viral trajectory dipped under the detection limit around 1.2 months (0.1 years), while M2 predicts the time of viral suppression at a time around 2.4 months (0.2 years). Nevertheless, both models predict ART initiation at around the time when the observed viral load is recorded, which is reasonable due to the occurrence of viral suppression.

For IDs 5 and 6, there are two fully observed viral loads before viral suppression. The values and the positions of the two viral loads influenced the shape of the entire trajectory. For example, compared to the slope of ID 6, the slope of the two viral loads for ID 5 is relatively flat; therefore, both M1 and M2 predict a flat pre-change point line.

We present the example for IDs 7, 8, and 9, where more fully observed viral loads are available. Here again, our model fits those data points quite well, and the predicted ART initiation times are reasonable. Importantly, ID 7 represents cases where ART initiation time is estimated to be zero, the time of HIV diagnosis. In such cases, the random change point model effectively degenerates to an NLME model. Similarly, ID 3 represents a case in which the change point model degenerates to an LME model, where the change occurs beyond the last observed viral load.

Visual inspection of Figure 3 reveals that the ART initiation times estimated by the ${\text{log}1\text{plus}}^{*}$ method generally align with the model-based predictions (M1 and M2) when viral load measurements show a sharp and well-timed decline (e.g., IDs 4, 5, and 7). However, notable discrepancies occur in cases with sparse measurements or censoring. For instance, in ID 6 and ID 8, the ${\text{log}1\text{plus}}^{*}$ method identifies ART initiation earlier than both M1 and M2, likely due to its reliance on observed declines rather than inferred trajectories. These differences underscore the advantage of the model-based approach in accommodating censoring, nonlinear post-ART dynamics, and between-subject variability in estimating ART initiation timing.

## 5. Simulation Studies

The performance of the StEM algorithm was evaluated through simulation. Here, we design two simulation studies aimed at assessing and comparing their estimation accuracy under different specifications for the post-ART segment, inspired by the real data analysis.

We model irregular viral load recording times since HIV diagnosis using a progressive state-transition model, assuming a first-order Markov process. This means that the length of time between viral records depends on the previous recording time. To generate a stochastic measurement time $\left(t_{i1},t_{i2},\ldots ,t_{in_{i}}\right)$, we use parameters obtained from fitting the model to the actual viral load test dates in HCCD. Specifically, we assume that the viral load measurement time T follows an exponential distribution with parameter ξ>0. Given the previous recording time u>0, the next recording time, conditioned on u, is given by $T_{|u}=-\frac{1}{\xi }\text{log}\;(X)+u$, where X~Uniform(0,1). For this simulation, we set ξ=1.6, which is estimated from the real data. The recording time is terminated when the time exceeds 3 years to simulate the real data. For left-censoring, in addition to simulating the 60% as in real data, we also run the model without any censoring to assess the StEM algorithm under such an ideal scenario.

To efficiently manage computational resources, each simulated dataset included 1000 individuals. In order to thoroughly evaluate the accuracy and bias of the estimated parameter values, we conducted 200 simulation runs for each scenario. Across these simulation replicates, we calculated both the mean squared error (MSE) and the bias (Bias) by comparing the estimated parameter values with the true values: $\text{Bias}=\frac{1}{S}\sum_{s=1}^{S}\;\left({\overset{ˆ}{\theta }}^{\left(s\right)}-\theta^{\text{true}}\right),\;\text{MSE}=\frac{1}{S}\sum_{s=1}^{S}\;100\times \frac{\sqrt{{\left({\overset{ˆ}{\theta }}^{\left(s\right)}-\theta^{\text{true}}\right)}^{2}+\text{SE}({\overset{ˆ}{\theta }}^{\left(s\right)})}}{\left|\theta^{\text{true}}\right|}$. Here, S is the number of replicates, and ${\overset{ˆ}{\theta }}^{\left(s\right)}$ is the estimate from simulation s.

Tables 2 and 3 display the simulation results for studies 1 and 2, respectively. We see that there is no major performance difference between M1 and M2. For each model, the fixed effects are estimated better than the dispersion parameters in general. The largest MSE occurred in the estimation of $\sigma_{A}^{2}$ under M1 when 60% of viral loads are left-censored. For M2, we see $\sigma_{B11}^{2}$ is estimated with the largest bias, while $\beta_{4}$, the second-phase decay rate, is estimated with the biggest MSE. Such sub-optimal performances are likely due to the sparsity of the observation frequency where insufficient data are available to provide the best estimation.

## 6. Conclusions and Discussion

In this paper, we extend the StEM algorithm by incorporating a combination of Gibbs sampling and Metropolis–Hastings steps to address the complexity of modeling individual-specific change points in longitudinal data. Specifically, we model change points using a zero-inflated exponential (ZIE) distribution, allowing us to capture both immediate and delayed antiretroviral therapy (ART) initiation. We also generalize standard random change point models by incorporating nonlinear mixed-effects (NLME) specifications before and after the change point.

Although our algorithm uses MCMC-based imputation steps reminiscent of Bayesian methods, it is embedded within a maximum likelihood framework. This hybrid structure avoids full posterior sampling—particularly of high-dimensional variance components—resulting in improved computational efficiency and estimation stability. It also preserves desirable asymptotic properties of maximum likelihood estimators while allowing for flexible inference via stochastic approximation. In addition, although our primary emphasis has been on point estimation, the framework supports approximate inference via a score-based variance-covariance estimator, enabling the construction of confidence intervals and facilitating hypothesis testing for model parameters.

Our method is evaluated through simulation studies and real data analysis. Compared to empirical rule-based approaches, which often suffer from instability due to measurement error or sparse sampling, our model-based framework leverages the full structure of the data to yield more reliable estimates. Application to clinical HIV cohort data demonstrates the utility of the method, revealing that a substantial proportion of individuals initiate ART immediately upon diagnosis—a biologically meaningful finding in light of “test-and-treat” policies.

Beyond statistical modeling, the clinical context of ART initiation is critical. While immediate initiation is now the recommended standard, delays remain common due to factors such as patient hesitancy, anticipated side effects, co-occurring mental health or substance use issues, stigma, or gaps in follow-up care. By accommodating both immediate and delayed ART initiation, the zero-inflated change point model enhances public health relevance and interpretability.

On the computational side, our use of Metropolis-within-Gibbs sampling and Geweke diagnostics enables convergence within 3000 iterations, even for complex, high-dimensional missing data structures. While our framework is flexible enough to incorporate other zero-inflated distributions (e.g., gamma, Weibull, or log-normal), we limited this work to the exponential case to preserve computational tractability. Future work will explore more efficient variants of the algorithm, such as independent sample approaches [32], to accommodate these extensions.

Several methodological developments offer promising directions. One is the integration of machine learning (ML) techniques—for example, to model post-ART viral dynamics or improve scalability. While ML algorithms may enhance flexibility, they often lack the interpretability and inferential tools required for surveillance-oriented estimation tasks. Hybrid approaches combining ML with structured statistical models could offer the best of both worlds and merit future study.

Another important extension involves incorporating subject-level covariates (e.g., age, gender, or transmission risk) into the fixed effects components of the model. This would allow parameters such as $\alpha_{1},\;\pi ,\;\lambda ,\;\beta_{1},\;\beta_{2}$, and $\beta_{3}$ to vary across individuals. However, this generalization would require replacing the closed-form M-step with iterative procedures such as Newton–Raphson, significantly increasing computational demands—particularly in the presence of censoring and complex random effects. We are actively pursuing this extension using parallel computing strategies to support large-scale applications.

It will also be important to assess the robustness of our method under misspecified change point distributions. While we focused on the ZIE distribution here, the algorithm can, in principle, accommodate zero-inflated log-Gaussian, gamma, or Weibull alternatives, as mentioned above. Incorporating these would, however, increase the complexity of Metropolis–Hastings updates and reduce sampling efficiency. Simulation-based robustness assessments under alternative distributions are planned as the next step.

## Figures and Tables

**Table 1.** Estimates in fitting HCCD data to the StEM algorithm with one-compartment (M1) and two-compartment (M2) specification for the post-ART trajectory.

Method	Parameter	α	$\beta_{1}$	$\beta_{2}$	$\beta_{3}$	$\beta_{4}$	π	λ
M1	est	0.43	10.51	3.92	1.69		0.31	1.11
	se	0.02	0.04	0.02	0.02		0.02	0.04
M2	est	0.42	10.52	3.89	2.72	−0.10	0.32	1.09
	se	0.02	0.03	0.02	0.02	0.07	0.02	0.04

Estimates of the variance components: $\text{M}1:\;\sigma_{A}^{2}=0.49,\left(\sigma_{B11}^{2},\sigma_{B22}^{2},\sigma_{B33}^{2}\right)=(4.61,0.64,1.92),\sigma_{e}^{2}=0.10$, $\text{M}2:\;\sigma_{A}^{2}=0.34,\left(\sigma_{B11}^{2},\sigma_{B22}^{2},\sigma_{B33}^{2},\sigma_{B44}^{2}\right)=(4.23,0.80,0.70,0.50),\sigma_{e}^{2}=0.11$,

**Table 2.** Simulation results on the performance of StEM algorithm when the post-ART trajectory is modeled with one-compartment model (M1).

θ	α	$\beta_{1}$	$\beta_{2}$	$\beta_{3}$	$\sigma_{A}^{2}$	$\sigma_{B11}^{2}$	$\sigma_{B22}^{2}$	$\sigma_{B33}^{2}$	$\sigma_{e}^{2}$	π	λ
True	0.43	10.51	3.92	1.69	0.49	4.61	0.64	1.92	0.10	0.31	1.11
0% left-censored											
Est	0.36	10.40	3.92	1.67	0.57	5.27	0.65	1.98	0.11	0.32	1.03
MSE	0.40	0.02	0.02	0.08	0.40	0.20	0.25	0.14	0.29	0.31	0.16
Bias	−0.7	−0.11	0.00	−0.02	0.08	0.66	0.01	0.06	0.03	0.01	−0.11
60% left-censored											
Est	0.39	10.38	4.02	2.01	1.20	6.59	0.76	1.34	0.14	0.34	0.99
MSE	0.49	0.03	0.04	0.26	1.50	0.45	0.35	0.52	0.43	0.31	0.17
Bias	−0.04	−0.13	0.10	0.32	0.71	1.98	0.12	−0.58	0.05	−0.02	−0.09

**Table 3.** Simulation results on the performance of StEM algorithm when the post-ART trajectory is modeled with two-compartment model (M2).

θ	α	$\beta_{1}$	$\beta_{2}$	$\beta_{3}$	$\beta_{4}$	$\sigma_{A}^{2}$	$\sigma_{B11}^{2}$	$\sigma_{B22}^{2}$	$\sigma_{B33}^{2}$	$\sigma_{B44}^{2}$	$\sigma_{e}^{2}$	π	λ
True	0.42	10.52	3.89	2.72	−0.10	0.34	4.23	0.80	0.70	0.50	0.11	0.32	1.09
0% left-censored													
Est	0.38	10.39	3.87	2.68	−0.47	0.37	5.82	0.90	1.42	0.50	0.12	0.32	1.07
MSE	0.33	0.03	0.04	0.05	0.39	0.39	0.44	0.23	1.14	0.25	0.56	0.33	0.18
Bias	−0.04	−0.13	−0.02	−0.04	−0.30	0.03	1.59	0.01	0.72	0.00	0.07	−0.00	−0.02
60% left-censored													
Est	0.42	10.23	3.78	2.23	−0.57	0.97	8.16	1.08	1.06	1.00	0.15	0.29	1.15
MSE	0.45	0.04	0.05	0.60	3.50	1.93	0.94	0.32	0.83	1.25	0.57	0.34	0.21
Bias	0.00	−0.29	−0.11	−1.79	−1.80	0.63	3.93	0.25	0.42	0.59	0.09	−0.03	0.06
